# Effects of Gender of Reciprocal Chromosomal Translocation on Blastocyst Formation and Pregnancy Outcome in Preimplantation Genetic Testing

**DOI:** 10.3389/fendo.2021.704299

**Published:** 2021-07-21

**Authors:** Hui Song, Hao Shi, En-tong Yang, Zhi-qin Bu, Zi-qi Jin, Ming-zhu Huo, Yi-le Zhang

**Affiliations:** ^1^ Reproductive Medicine Center, First Affiliated Hospital of Zhengzhou University, Zhengzhou, China; ^2^ Henan Key Laboratory of Reproduction and Genetics, First Affiliated Hospital of Zhengzhou University, Zhengzhou, China

**Keywords:** biopsy, clinical pregnancy rate, maternal age, paternal age, preimplantation genetic testing, reciprocal translocation, blastocyst formation rate, aeuploidy rate

## Abstract

**Objective:**

To determine the effect of gender of reciprocal chromosomal translocation on blastocyst formation and pregnancy outcome in preimplantation genetic testing, including different parental ages.

**Methods:**

This was a retrospective cohort study that enrolled 1034 couples undergoing preimplantation genetic testing-structural rearrangement on account of a carrier of reciprocal chromosomal translocation from the Reproductive Medicine Center of the First Affiliated Hospital of Zhengzhou University from January 2015 to December 2019. Group A represented 528 couples in which the man was the carrier of reciprocal translocation and group B represented 506 couples in which the woman was the carrier of reciprocal translocation. All patients were divided into two groups according to their age: female age<35 and female age≥35. Furthermore, the differences in blastocyst condition and pregnancy outcome between male and female carriers in each group were further explored according to their father’s age.

**Results:**

The blastocyst formation rate of group A (55.3%) is higher than that of group B (50%) and the results were statistically significant (P<0.05). The blastocyst formation rate of group A is higher than that of group B, no matter in young maternal age or in advanced maternal age (P<0.05). The blastocyst formation rate in maternal age<35y and paternal age<30y in group A(57.1%) is higher than that of Group B(50%); Similarly, the blastocyst formation rate in maternal age≥35 and paternal age≥38y(66.7%) is higher than that of Group B(33.3%)(all P<0.05). There was no difference in fertilization rate, aeuploidy rate, clinical pregnancy rate, miscarriage rate and live birth rate between Group A and Group B.

**Conclusion:**

When the carrier of reciprocal translocation is male, the blastocyst formation rate is higher than that of female carrier. While there is no significant difference between the two in terms of fertilization rate, aeuploidy rate, clinical pregnancy rate, miscarriage rate and live birth rate.

## Introduction

Preimplantation genetic testing (PGT) is a set of advanced clinical procedures that includes PGT for aneuploidies (PGT-A) and PGT for chromosomal structural rearrangements (PGT-SR) (1). It is a method for testing *in vitro* embryos or oocytes for Mendelian, chromosomal and mitochondrial defects as an alternative for pre-natal diagnosis and selective termination of pregnancy in couples with a high risk of affected offspring, and many different genetic diseases can be diagnosed including structural chromosome rearrangements such as translocations (2). The primary aim of preimplantation genetic testing-structural rearrangement (PGT-SR) would be to distinguish between embryos carrying the “reciprocal translocation” (and accompanying microdeletion) and those with truly normal chromosomes (3). Couples with genetic diseases can choose PGT to increase their chances of pregnancy and obtain healthy offspring (4, 5).

Chromosome reciprocal translocation is a common chromosome structural abnormality (6). It is estimated that 1 in 625 individuals carries a reciprocal translocation (3). Since there is no increase or decrease in chromosomal fragment information, the carrier of reciprocal translocation has a normal phenotype (7). However, It is possible that the presence of an unreciprocal translocation in some gametes of translocation carriers may result in failure of the developing embryo to implant or in early pregnancy loss (8). The theoretical chance of producing normal or reciprocal gametes is 4 of 32 for reciprocal translocations. However, the actual outcome depends on many factors, including the chromosomes involved, the breakpoints, and the sex of the carrier (9).

In this study, couples who underwent PGT-SR assisted pregnancy were grouped and compared according to the gender of the reciprocal translocation carriers to analyze the effect of reciprocal translocation of different sex chromosomes on blastocyst formation and pregnancy outcome, including different parental ages. Therefore to determine the relationship between gender and age of reciprocal translocation carriers and blastocyst formation and pregnancy outcome in couples undergoing PGT-SR assisted pregnancy.

## Materials and Methods

### Study Population

This study is a retrospective analysis of PGT-SR cycles performed at the Reproductive Medicine Center of the First Affiliated Hospital of Zhengzhou University from January 2015 to December 2019. Inclusion criteria were as follows: 1. PGT-SR cycles performed at the Reproductive Medicine Center of the First Affiliated Hospital of Zhengzhou University from January 2015 to December 2019. 2. One and only one of both parents is a carrier of reciprocal translocation. 3. Total number of eggs obtained ≥3 (10).

### Grouping

According to the gender of the reciprocal translocation carriers, the patients were divided into two groups. Group A: Male with reciprocal chromosomal translocation and female with normal chromosome karyotype; Group B: Female with reciprocal chromosomal translocation and male with normal chromosomal karyotype (**Table 1**). Then patients were allocated into two maternal age groups (11, 12) (<35、≥35y) (**Table 2**), three paternal age groups (13)(≤30、30-34、≥35y) and two paternal age groups (14) (<38、≥38y) called A1-A5 and B1-B5 (**Table 3**). The main outcomes were blastocyst formation rate and clinical pregnancy rate, and the secondary outcomes were fertilization rate, aeuploidy rate, miscarriage rate and live birth rate.

**Table 1:** Comparison of overall conditions between male and female carriers.

Items	Group A	Group B	P-value
**PGD cycles**	528	506	
**Male age (y)**	29.0 ± 5.0	29.0 ± 6.0	0.109
**Female age (y)**	29.0 ± 5.0	29.0 ± 6.0	0.376
**Retrieved oocytes**	17.0 ± 11.0	16.0 ± 11.0	0.262
**MII**	14.0 ± 10.0	14.0 ± 10.0	0.221
**Normal fertilization (2PN)**	11.0 ± 9.0	11.0 ± 8.0	0.315
**Fertilization rate (%)**	68.0 ± 24.0	68.0 ± 24.0	0.874
**Embryo biopsied**	5.0 ± 5.0	4.0 ± 4.0	0.068
**Aeuploidy rate (%)**	77.8 ± 40.0	75.0 ± 44.0	0.594
**Blastocyst formation rate (%)**	55.3 ± 35.0	50.0 ± 37.0	0.001*
**Clinical pregnancy rate (%)**	56.7 (148/261)	57.5 (134/233)	0.857
**Miscarriage rate (%)**	10.7 (28/261)	9.4 (22/233)	0.636
**Live birth rate (%)**	45.6 (119/261)	47.6 (111/233)	0.649

Group A, couple with a male carrier; Group B, couple with a female carrier.

() represents the number of positives to total.

*P < 0.05 was considered statistically significant.

**Table 2:** Blastocyst conditions and pregnancy outcomes between male carriers and female carriers in young and advanced maternal age.

Items	female age<35			female age≥35		
	Group A	Group B	P-value	Group A	Group B	P-value
**Fertilization rate (%)**	68.0 ± 24.0	66.7 ± 25.0	0.895	67.7 ± 17.6	69.6 ± 16.0	0.55
**Aeuploidy rate (%)**	77.8 ± 40.0	75.0 ± 43.0	0.461	75.0 ± 50.0	80.0 ± 50.0	0.657
**Blastocyst formation rate (%)**	53.9 ± 34.0	50.0 ± 37.0	0.021*	60.0 ± 42.0	40.0 ± 35.0	0.003*
**Clinical pregnancy rate (%)**	56.3 (135/240)	57.1 (124/217)	0.847	61.9 (13/21)	62.5 (10/16)	0.970
**Miscarriage rate (%)**	10.8 (26/240)	9.7 (21/217)	0.685	9.5 (2/21)	6.3 (1/16)	0.718
**Live birth rate (%)**	45.0 (108/240)	47.0 (102/217)	0.668	52.4 (11/21)	56.3 (9/16)	0.815

Group A, couple with a male carrier; Group B, couple with a female carrier.

() represents the number of positives to total.

*P < 0.05 was considered statistically significant.

**Table 3:** Comparison of blastocyst conditions between male and female carriers in different age groups.

Items	Group A	Group B	P-value
**maternal age<35**			
**① paternal age<30**			
PGD cycles	281 (A1)	256 (B1)	
Fertilization rate (%)	68.8 ± 24.0	66.7 ± 25.0	0.975
Blastocyst formation rate (%)	57.1 ± 31.0	50.0 ± 36.0	0.003*
Aeuploidy rate (%)	80.0 ± 38.0	75.0 ± 48.0	0.036*
**② paternal age 30-34**			
PGD cycles	153 (A2)	143 (B2)	
Fertilization rate (%)	66.9 ± 16.4	67.7 ± 17.4	0.546
Blastocyst formation rate (%)	50.0 ± 33.0	50.0 ± 38.0	0.895
Aeuploidy rate (%)	75.0 ± 48.0	80.0 ± 33.0	0.057
**③ paternal age≥35**			
PGD cycles	39 (A3)	48 (B3)	
Fertilization rate (%)	65.8 ± 15.8	61.4 ± 16.2	0.205
Blastocyst formation rate (%)	47.0 ± 26.3	46.5 ± 25.4	0.925
Aeuploidy rate (%)	80.0 ± 38.0	75.0 ± 49.0	0.421
**maternal age≥35**			
**④ paternal age<38**			
PGD cycles	28 (A4)	27 (B4)	
Fertilization rate (%)	68 ± 17.4	68.6 ± 15.5	0.884
Blastocyst formation rate (%)	50.0 ± 51.0	42.9 ± 27.0	0.199
Aeuploidy rate (%)	80.9 ± 48.0	75.0 ± 50.0	0.897
**⑤ paternal age≥38**			
PGD cycles	27 (A5)	32 (B5)	
Fertilization rate (%)	67.4 ± 18.2	70.4 ± 16.6	0.513
Blastocyst formation rate (%)	66.7 ± 39.0	33.3 ± 47.0	0.003*
Aeuploidy rate (%)	75.0 ± 50.0	81.7 ± 50.0	0.624

Group A, couple with a male carrier; Group B, couple with a female carrier. A1, maternal age < 35y and paternal age < 30y in Group A; B1, maternal age < 35y and paternal age < 30y in Group B; A2, maternal age < 35y and paternal age between 30-34y in Group A; B2, maternal age < 35y and paternal age between 30-34y in Group B; A3, maternal age < 35y and paternal age ≥ 35y in Group A; B3, maternal age < 35y and paternal age ≥ 35y in Group B; A4, maternal age ≥ 35y and paternal age < 38y in Group A; B4, maternal age ≥ 35y and paternal age ≥ 38y in Group B; A5, maternal age ≥ 35y and paternal age ≥ 38y in Group A; B5, maternal age ≥ 35y and paternal age ≥ 38y in Group B. The clinical pregnancy rate, miscarriage rate and live birth rate were not given as before due to limitation of the number of couples who had pregnancy outcomes within each small group.

*P < 0.05 was considered statistically significant.

### Ovulation-Inducing

Subcutaneous injection of short-acting gonadotropin-releasing hormone agonist (0.05-0.1 mg of Diphereline, Ipsen, Paris, France) was performed once a day in the mid-luteal phase of the previous cycle. After complete pituitary down regulation, gonadotrophin (Gn), a high-purity recombinant follicle stimulating hormone (r-FSH, Merck Serono SPA, Geneva, Switzerland, or HMG, Le Baode, Shanghai Lizhu Pharmaceutical Co. Ltd., Shanghai, China), was used until HCG day. Gn dose was adjusted according to patients’ specific conditions. Oocyte retrieval, insemination, embryo transfer and corpus luteum support were performed according to our routine protocols (15, 16).

### Embryo Culture and Biopsy

Intracytoplasmic sperm injection was performed in 4 to 6 h after retrieval for all cycles. The embryos were cultured to the blastocyst stage of 5-6 days (according to the procedures of intracytoplasmic single sperm microinjection and embryo culture of our center), and blastocysts with the grade of 3BC or above were selected for biopsy (blastocysts that did not reach the grade of biopsy were scrapped after the patient signed the informed consent). Three to five trophectoderm cells were aspirated with biopsy needles combined with laser-assisted cleavage. Whole genome DNA of biopsied trophectoderm cell was amplified and SNP microarray chip detection technology (SNP array) or next-generation sequencing (NGS) technology were used to detect aneuploidy (17–19). The blastocysts after biopsy were quickly transferred to a four-well plate and placed in an incubator at 37°C and 6% CO2 for freezing.

### Frozen and Thawed Blastocyst Transplantation

After biopsy, the blastocysts were vitrified and stored in liquid nitrogen. On transplantation day, frozen blastocysts were removed, thawed and transplanted into culture medium (6%HSA G-2) for 2h before transplantation. Blood HCG was measured 14 and 18 days after embryo transfer and abdominal ultrasonography was performed 35d after transplantation. Ultrasound showing a gestational sac was regarded as clinical pregnancy. Blastocyst formation rate = number of blastocysts formed/number of cultured blastocysts (number of 2PN fertilized eggs from D3 cultured blastocysts) ×100% (20); Clinical pregnancy rate = clinical pregnancy number/transplantation cycle number ×100%.

### Statistical Analysis

Statistical analysis was performed using SPSS 21.0 software. The measurement data conforming to normal distribution were tested by two independent samples to test and the results were presented as mean ± standard deviation; the measurement data not conforming to normal distribution were tested by rank sum test and the results were presented as median ± quartile spacing; the counting data were tested by chi-square test. The results of the rate were presented as a percentage (%). P <0.05 was considered to indicate statistical significance.

## Results

### Comparison of the General Characteristics Between Male Carriers and Female Carriers

**Table 1** shows the general characteristics for both sets of data. Group A: Male with reciprocal chromosomal translocation and female with normal chromosome karyotype; Group B: Female with reciprocal chromosomal translocation and male with normal chromosomal karyotype. A total of 1034 PGT-SR cycles were included with 528 couples in Group A and 506 couples in Group B. Overall, there was no significant difference in male age, female age, retrieved oocytes, MII number of eggs, normal fertilization, fertilization rate, embryo biopsied, aeuploidy rate, clinical pregnancy, miscarriage rate and live birth rate between Group A and Group B. However, our data showed that the blastocyst formation rate in Group A is 55.3%, which is higher than that in Group B, whose blastocyst formation rate is 50.0%, and the results were statistically significant (P<0.05).

### Main Outcomes of the Two Groups in Young and Advanced Maternal Age

To determine the influence of young maternal age and advanced maternal age on the results of the comparison between the two groups, we divided the maternal age into two groups: <35y and ≥35y, and compared the blastocyst formation and pregnancy outcome of A and B in each group. The comparison results are shown in **Table 2**. According to the data in the table, we found that the blastocyst formation rate of group A was 53.9%, which was still higher than that of group B (50.0%) in couples whose maternal age<35. While there was no difference in fertilization rate, aeuploidy rate, clinical pregnancy rate, miscarriage rate and live birth rate. Similarly, among couples with a maternal age≥35, the blastocyst formation rate in group A was 60.0%, which was higher than that in group B (40.0%), but there was no difference in fertilization rate, aeuploidy rate, clinical pregnancy rate, miscarriage rate and live birth rate.

### Influence of Paternal Age on Comparison Between Male Carriers and Female Carriers

**Figure 1:**
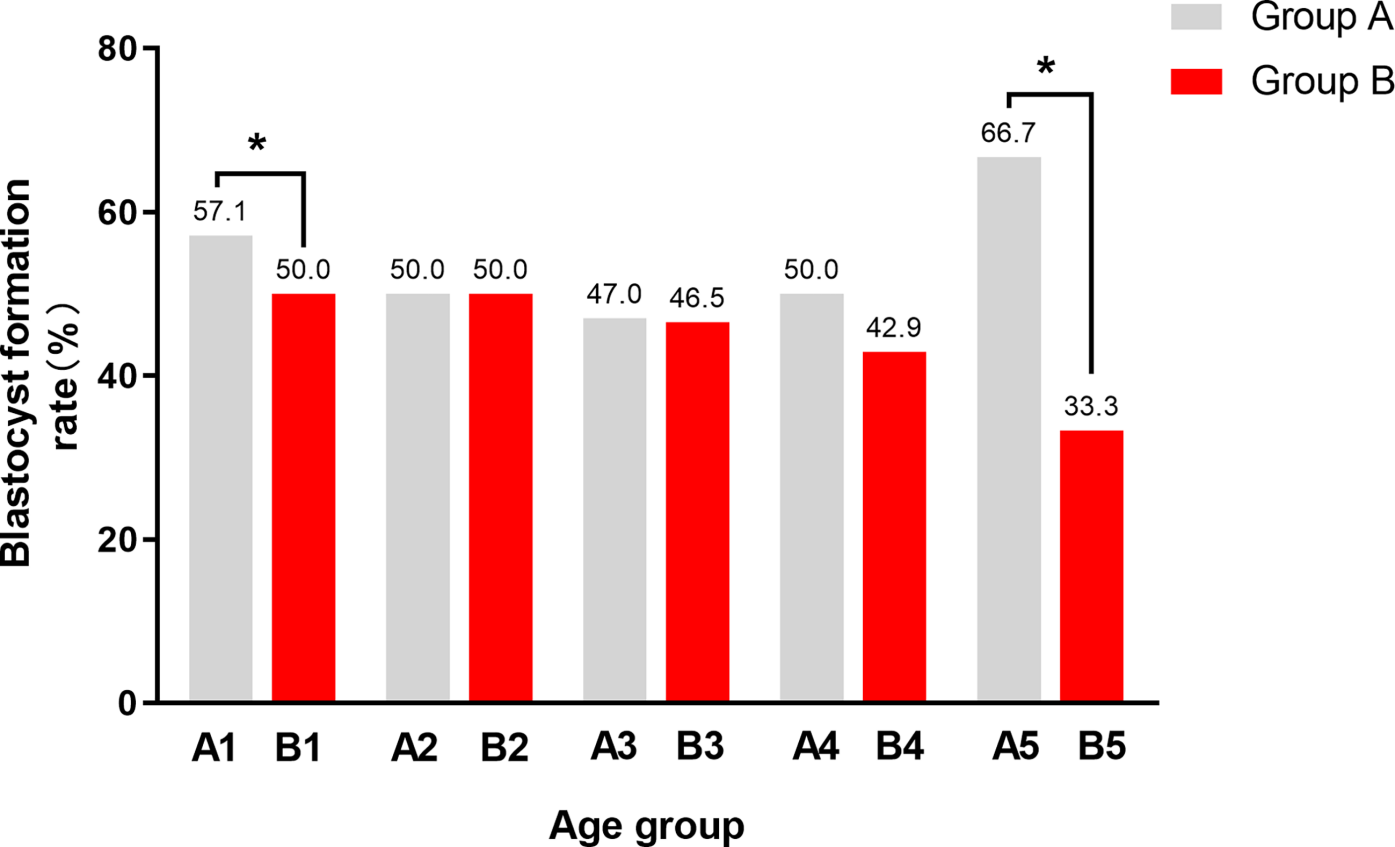
The blastocyst formation rate between male carriers and female carriers in different age groups. Group A, couple with a male carrier; Group B, couple with a female carrier. A1, maternal age < 35y and paternal age < 30y in Group A; B1, maternal age < 35y and paternal age < 30y in Group B; A2, maternal age < 35y and paternal age between 30-34y in Group A; B2, maternal age < 35y and paternal age between 30-34y in Group B; A3, maternal age < 35y and paternal age ≥ 35y in Group A; B3, maternal age < 35y and paternal age ≥ 35y in Group B; A4, maternal age ≥ 35y and paternal age < 38y in Group A; B4, maternal age ≥ 35y and paternal age ≥ 38y in Group B; A5, maternal age ≥ 35y and paternal age ≥ 38y in Group A; B5, maternal age ≥ 35y and paternal age ≥ 38y in Group **(B)** The most significant difference was between A5 and B5, whose maternal age ≥ 35 and paternal age ≥ 38. In addition, there was a statistical difference between A1 and B1,whose maternal age < 35 and paternal age < 30. The blastocyst formation rate of male carriers is higher than that of female carriers in both 1 and 5 groups. *P < 0.05 was considered statistically significant.

Further, in order to explore the influence of paternal age on blastocyst formation, we classified paternal age according to ≤ 30, 30-34, ≥35 years old and<38, ≥38 years old, as is shown in **Table 3**. The fertilization rate, blastocyst formation rate, and aeuploidy rate of group A and group B were compared in different age groups and the comparison results are shown in **Table 3**. Among couples with maternal age<35y and paternal age<30y, the blastocyst formation rate of Group A is 57.1%, which is higher than that of Group B, whose blastocyst formation rate is 50.0%. Similarly, Among couples with maternal age≥35y and paternal age≥38y, the blastocyst formation rate of Group A is 66.7%, which is higher than that of Group B, whose blastocyst formation rate is 33.3%. For the rest (**Figure 1**), there is no significant difference on blastocyst formation rate in other age groups between A and B, and there is no statistical difference on fertilization rate or aeuploidy rate in any age groups between A and B.

## Discussion

We report the real data of PGT-SR performed in the First Affiliated Hospital of Zhengzhou University from 2013 to 2019 due to one parent suffering from reciprocal chromosomal translocation. In the comparison of overall characteristics, we found that in the case of the same female age, male age, retrieved oocytes, MII number of eggs, normal fertilization, aeuploidy rate and fertilization rate, the blastocyst formation rate in male carriers was higher than that of female carriers. While there was no difference in pregnancy outcomes between the two groups. After comparing the young maternal age group with the advanced maternal age group, we came to the same conclusion that if the reciprocal translocation occurs in the male, the blastocyst formation rate is higher and there was no difference in pregnancy outcomes. This is consistent with K. Haapaniemi Kouru’s finding that in the reciprocal translocation group, the couple was more likely to conceive if it was the man who was the carrier of the translocation (21). On the contrary, Kort JD found that patients with an indication of male factor infertility having significantly fewer blastocysts available for biopsy than maternal age-matched cohorts with different indications (22). Our research indicates that what he said “male factor” may not include “a carrier of reciprocal chromosomal translocation”. In the further comparison, we can see that in the age group of maternal age<35 and paternal age<30, the blastocyst formation rate of couples in male carriers is higher than that of female carriers. And the same result also occurred in the age group of maternal age≥35 and paternal age≥38, what comes to a consistent conclusion with the general comparison in male carriers and female carriers. But for the fertilization rate, aeuploidy rate, clinical pregnancy rate, miscarriage rate and live birth rate, there was no statistical difference between the male carriers and the female carriers. Hence we encourage couples no matter the male or the female is a carrier of reciprocal translocation to underdo PGT-SR assisted fertility treatment, because there is no difference in clinical pregnancy outcomes between the two.

Regarding the aeuploidy rate, our data showed no difference between the male disease group(77.8%) and the female disease group(75%), which is consistent with the results of Belén Lledó and his group that the proportions of alternate segregation for normal or reciprocal chromosome contents in preimplantation embryos from PGD cycles in reciprocal-male and female carriers were not significantly different (23). In A study of 1800 IVF cycles involving 3117 blastocyst biopsies, the rate of aneuploidy of PGT-A was 42.9% (24). While that of PGT-SR in our research reach up to 77.8%, from which we can see that the detection rate of aneuploidy of PGT-SR is higher than that of PGT-A. Up to now, the impact of PGS on the outcome of assisted reproduction remains uncertain and needs for larger, high quality trials (25). At present, PGT has become a basic and important choice of assisted reproductive technology for patients with chromosomal translocations. With the continuous improvement and development of technology, more and more chromosome detection methods have been applied in PGT. The cases in this article all use the SNP or NGS method, which is currently a relatively extensive method for the diagnosis of embryo chromosome abnormality (19). They can screen 23 pairs of chromosomes comprehensively, which has obvious advantages compared with FISH technique which can only carry out hybridization detection of a limited number of chromosomes (26). What is more, trophoblastic cell biopsy can not only control the damage to the embryo, but also ensure the accuracy of the sequencing results (27).

Blastocyst culture is a process of further screening of embryos. The blastocyst formation rate is closely related to the normality of the embryo’s chromosomes which come from both parents. During meiosis, the two translocated chromosomes and their two homologous normal chromosomes form a quadrivalent and subsequently segregate at anaphase I. Normal or reciprocal gametes are produced by an alternate mode of segregation. Gametes produced by the other segregation patterns have unreciprocal karyotypes (28, 29). The effect of a carrier’s sex on meiotic segregation of the quadrivalent has been investigated previously, with some studies reporting that sex differentially affected the meiotic segregation patterns (29, 30). Studies have suggested that there are many factors that affect the separation of chromosomes in meiosis, including the gender of carriers, maternal age, the type of translocated chromosomes and the length of translocated fragments (31, 32). The nature of oogenesis is such that female gametes are more susceptible to meiotic chromosome errors, compared to those of the male (33). This is consistent with the conclusion of this study. The main purpose of this study was to analyze the influence of gender of reciprocal chromosomal translocation on blastocyst formation and pregnancy outcome in preimplantation genetic diagnosis, and to further explore the influence of parental age on it.

## Conclusions

When the carrier of reciprocal translocation is male, the blastocyst formation rate is higher than that of female carrier. While there was no significant difference between the two in terms of fertilization rate, aeuploidy rate, clinical pregnancy rate, miscarriage rate and live birth rate. Hence we encourage couples no matter the male or the female is a carrier of reciprocal translocation to undergo PGT-SR assisted fertility treatment, because there was no difference in clinical pregnancy outcomes between the two.

### Clinical Importance

In recent years, there are many studies on the relationship between embryonic chromosomal abnormality, aneuploidy rate and parental age (34). While, studies on the relevance of embryonic development and gender of reciprocal translocation, parental age is rare. Therefore, our data not only provide a basis for the age of undergoing PGT for couples suffering from reciprocal translocation in clinical practice, but also triggers thinking about the potential connection between the occurrence of reciprocal translocation in couples and blastocyst formation, and the specific mechanism is worthy of further explore.

### Strengths and Limitations

The main strength of our study was that we performed a statistical analysis of a large sample of patients with reciprocal chromosomal translocations. In addition, this was an original study. However, our study also had several limitations due to its retrospective design and a single medical center, and there is an inevitable bias. In addition, this research is only a preliminary discussion and the conclusions needed to be interpreted carefully.

## Data Availability Statement

The original contributions presented in the study are included in the article/supplementary material. Further inquiries can be directed to the corresponding author.

## Author Contributions

All authors listed have made a substantial, direct, and intellectual contribution to the work, and approved it for publication.

## Funding

This project was supported by grant 31970799 from the National Natural Science Foundation of China.

## Conflict of Interest

The authors declare that the research was conducted in the absence of any commercial or financial relationships that could be construed as a potential conflict of interest.

## References

[1] Li G Wu YF Niu WB Xu JW Hu LL Shi H. Analysis of the Number of Euploid Embryos in Preimplantation Genetic Testing Cycles With Early-Follicular Phase Long-Acting Gonadotropin-Releasing Hormone Agonist Long Protocol. Front Endocrinol (Lausanne) (2020) 11:424. doi: 10.3389/fendo.2020.00424 32793112 PMC7386196

[2] Geraedts JP De Wert GM. Preimplantation Genetic Diagnosis. Clin Genet (2009) 76(4):315–25. doi: 10.1111/j.1399-0004.2009.01273.x 19793305

[3] Treff NR Tao X Schillings WJ Bergh PA Scott RT Jr Levy B. Use of Single Nucleotide Polymorphism Microarrays to Distinguish Between Balanced and Normal Chromosomes in Embryos From a Translocation Carrier. Fertil Steril (2011) 96(1):e58–65. doi: 10.1016/j.fertnstert.2011.04.038 21575938

[4] Kuliev A Rechitsky S. Preimplantation Genetic Testing: Current Challenges and Future Prospects. Expert Rev Mol Diagn (2017) 17(12):1071–88. doi: 10.1080/14737159.2017.1394186 29039978

[5] Wang YZ Ding CH Wang J Zeng YH Zhou W Li R. Number of Blastocysts Biopsied as a Predictive Indicator to Obtain at Least One Normal/Balanced Embryo Following Preimplantation Genetic Diagnosis With Single Nucleotide Polymorphism Microarray in Translocation Cases. J Assist Reprod Genet (2017) 34(1):51–9. doi: 10.1007/s10815-016-0831-0 PMC5330983 27822654

[6] Morin SJ Eccles J Iturriaga A Zimmerman RS. Translocations, Inversions and Other Chromosome Rearrangements. Fertil Steril (2017) 107(1):19–26. doi: 10.1016/j.fertnstert.2016.10.013 27793378

[7] Sampson JE Ouhibi N Lawce H Patton PE Battaglia DE Burry KA. The Role for Preimplantation Genetic Diagnosis in Balanced Translocation Carriers. Am J Obstet Gynecol (2004) 190(6):1707–11. doi: 10.1016/j.ajog.2004.02.063 15284776

[8] Flowers NJ Burgess T Giouzeppos O Shi G Love CJ Hunt CE. Genome-Wide Noninvasive Prenatal Screening for Carriers of Balanced Reciprocal Translocations. Genet Med (2020) 22(12):1944–55. doi: 10.1038/s41436-020-0930-2 32807973

[9] Rius M Obradors A Daina G Ramos L Pujol A Martínez-Passarell O. Detection of Unbalanced Chromosome Segregations in Preimplantation Genetic Diagnosis of Translocations by Short Comparative Genomic Hibridization. Fertil Steril (2011) 96(1):134–42. doi: 10.1016/j.fertnstert.2011.04.052 21596375

[10] Chapuis A Gala A Ferrières-Hoa A Mullet T Bringer-Deutsch S Vintejoux E. Sperm Quality and Paternal Age: Effect on Blastocyst Formation and Pregnancy Rates. Basic Clin Androl (2017) 27:2. doi: 10.1186/s12610-016-0045-4 28127436 PMC5251225

[11] Taylor TH Patrick JL Gitlin SA Crain JL Wilson JM Griffin DK. Blastocyst Euploidy and Implantation Rates in a Young (<35 Years) and Old (≥35 Years) Presumed Fertile and Infertile Patient Population. Fertil Steril (2014) 102:1318–23. doi: 10.1016/j.fertnstert.2014.07.1207 25154676

[12] Ubaldi FM Cimadomo D Vaiarelli A Fabozzi G Venturella R Maggiulli R. Advanced Maternal Age in IVF: Still a Challenge? The Present and the Future of Its Treatment. Front Endocrinol (Lausanne) (2019) 10:94. doi: 10.3389/fendo.2019.00094 30842755 PMC6391863

[13] Broekmans FJ Soules MR Fauser BC. Ovarian Aging: Mechanisms and Clinical Consequences. Endocr Rev (2009) 30(5):465–93. doi: 10.1210/er.2009-0006 19589949

[14] Wu Y Kang X Zheng H Liu H Liu J. Effect of Paternal Age on Reproductive Outcomes of *In Vitro* Fertilization. PloS One (2015) 10(9):e0135734. doi: 10.1371/journal.pone.0135734 26352861 PMC4564138

[15] Zhang YL Wang XY Wang F Su YC Sun YP. Clinical Analysis of Spontaneous Pregnancy Reduction in the Patients With Multiple Pregnancies Undergoing *In Vitro* Fertilization/Intracytoplasmic Sperm Injection-Embryo Transfer. Int J Clin Exp Med (2015) 8(3):4575–80. PMC4443221 26064387

[16] Zhang YL Sun J Su YC Guo YH Sun YP. Ectopic Pregnancy in Frozen-Thawed Embryo Transfer: A Retrospective Analysis of 4034 Cycles and Related Factors. Syst Biol Reprod Med (2013) 59:34–7. doi: 10.3109/19396368.2012.731470 23050806

[17] Bernstein LR Treff NR. Editorial: Causes of Oocyte Aneuploidy and Infertility in Advanced Maternal Age and Emerging Therapeutic Approaches. Front Endocrinol (Lausanne) (2021) 12:652990. doi: 10.3389/fendo.2021.652990 33708177 PMC7940751

[18] Poli M Girardi L Fabiani M Moretto M Romanelli V Patassini C. Past, Present, and Future Strategies for Enhanced Assessment of Embryo’s Genome and Reproductive Competence in Women of Advanced Reproductive Age. Front Endocrinol (Lausanne) (2019) 10:154. doi: 10.3389/fendo.2019.00154 30941103 PMC6433971

[19] Xu J Fang R Chen L Chen D Xiao JP Yang W. Noninvasive Chromosome Screening of Human Embryos by Genome Sequencing of Embryo Culture Medium for *In Vitro* Fertilization. Proc Natl Acad Sci USA (2016) 113(42):11907–12. doi: 10.1073/pnas.1613294113 PMC5081593 27688762

[20] Alpha Scientists in Reproductive Medicine and ESHRE Special Interest Group Embryology. Istanbul Consensus Workshop on Embryo Assessment: Proceedings of an Expert Meeting. Reprod BioMed Online (2011) 22(6):1270–83. doi: 10.1016/j.rbmo.2011.02.001 21481639

[21] Haapaniemi Kouru K Malmgren H Nordenskjöld M Fridström M Csemiczky G Blennow E. One-Cell Biopsy Significantly Improves the Outcome of Preimplantation Genetic Diagnosis (PGD) Treatment: Retrospective Analysis of 569 PGD Cycles at the Stockholm PGD Centre. Hum Reprod (2012) 27(9):2843–9. doi: 10.1093/humrep/des235 22736325

[22] Kort JD McCoy RC Demko Z Lathi RB. Are Blastocyst Aneuploidy Rates Different Between Fertile and Infertile Populations? J Assist Reprod Genet (2018) 35(3):403–8. doi: 10.1007/s10815-017-1060-x PMC5904052 29063503

[23] Lledó B Ortiz JA Morales R Ten J de la Fuente PE García-Ochoa C. The Paternal Effect of Chromosome Translocation Carriers Observed From Meiotic Segregation in Embryos. Hum Reprod (2010) 25(7):1843–8. doi: 10.1093/humrep/deq111 20511301

[24] Alexander LS Michelle K Erin F GP J Mark RB Carolyn G. Pregnancy Outcomes From More Than 1,800 *In Vitro* Fertilization Cycles With the Use of 24-Chromosome Single-Nucleotide Polymorphism-Based Preimplantation Genetic Testing for Aneuploidy. Fertil Steril (2018) 110(1):113–21. doi: 10.1016/j.fertnstert.2018.03.026 29908770

[25] Hodes-Wertz B Grifo J Ghadir S Kaplan B Laskin CA Glassner M. Idiopathic Recurrent Miscarriage Is Caused Mostly by Aneuploid Embryos. Fertil Steril (2012) 98(3):675–80. doi: 10.1016/j.fertnstert.2012.05.025 22683012

[26] Niu W Wang L Xu J Li Y Shi H Li G. Improved Clinical Outcomes of Preimplantation Genetic Testing for Aneuploidy Using MALBAC-NGS Compared With MDA-SNP Array. BMC Pregnancy Childbirth (2020) 20(1):388. doi: 10.1186/s12884-020-03082-9 32620095 PMC7333433

[27] Scriven PN Handyside AH Ogilvie CM. Chromosome Translocations: Segregation Modes and Strategies for Preimplantation Enetic Diagnosis. Prenat Diagn (1998) 18(13):1437–49. doi: 10.1002/(SICI)1097-0223(199812)18:13<1437::AID-PD497>3.0.CO;2-P 9949444

[28] Zhang L Wei D Zhu Y Jiang W Xia M Li J. Interaction of Acrocentric Chromosome Involved in Translocation and Sex of the Carrier Influences the Proportion of Alternate Segregation in Autosomal Reciprocal Translocations. Hum Reprod (2019) 34(2):380–7. doi: 10.1093/humrep/dey367 30576528

[29] Ko DS Cho JW Park SY Kim JY Koong MK Song IO. Clinical Outcomes of Preimplantation Genetic Diagnosis (PGD) and Analysis of Meiotic Segregation Modes in Reciprocal Translocation Carriers. Am J Med Genet A (2010) 152A(6):1428–33. doi: 10.1002/ajmg.a.33368 20503317

[30] Zhang S Lei C Wu J Sun H Zhou J Zhu S. Analysis of Segregation Patterns of Quadrivalent Structures and the Effect on Genome Stability During Meiosis in Reciprocal Translocation Carriers. Hum Reprod (Oxford England) (2018) 33(4):757–67. doi: 10.1093/humrep/dey036 29579270

[31] Ye Y Qian Y Xu C Jin F. Meiotic Segregation Analysis of Embryos From Reciprocal Translocation Carriers in PGD Cycles. Reprod Biomed Online (2012) 24(1):83–90. doi: 10.1016/j.rbmo.2011.08.012 22116068

[32] Fragouli E Munne S Wells D. The Cytogenetic Constitution of Human Blastocysts: Insights From Comprehensive Chromosome Screening Strategies. Hum Reprod Update (2019) 25(1):15–33. doi: 10.1093/humupd/dmy036 30395265

[33] Hassold T Hunt P. Maternal Age and Chromosomally Abnormal Pregnancies: What We Know and What We Wish We Knew. Curr Opin Pediatr (2009) 21(6):703–708. doi: 10.1097/MOP.0b013e328332c6ab 19881348 PMC2894811

[34] Franasiak JM Forman EJ Hong KH Werner MD Upham KM Treff NR. The Nature of Aneuploidy With Increasing Age of the Female Partner: A Review of 15,169 Consecutive Trophectoderm Biopsies Evaluated With Comprehensive Chromosomal Screening. Fertil Steril (2014) 101(3):656–663.e1. doi: 10.1016/j.fertnstert.2013.11.004 24355045

