# Corrigendum: Effects of Gender of Reciprocal Chromosomal Translocation on Blastocyst Formation and Pregnancy Outcome in Preimplantation Genetic Testing

**DOI:** 10.3389/fendo.2021.788988

**Published:** 2021-11-10

**Authors:** Hui Song, Hao Shi, En-tong Yang, Zhi-qin Bu, Zi-qi Jin, Ming-zhu Huo, Yi-le Zhang

**Affiliations:** ^1^ Reproductive Medicine Center, First Affiliated Hospital of Zhengzhou University, Zhengzhou, China; ^2^ Henan Key Laboratory of Reproduction and Genetics, First Affiliated Hospital of Zhengzhou University, Zhengzhou, China

**Keywords:** biopsy, clinical pregnancy rate, maternal age, paternal age, preimplantation genetic testing, reciprocal translocation, blastocyst formation rate, aeuploidy rate

There is an error in the **Funding** statement. The correct number for the National Natural Science Foundation of China grant is 31970799.

The authors apologize for this error and state that this does not change the scientific conclusions of the article in any way. The original article has been updated.

## Publisher’s Note

All claims expressed in this article are solely those of the authors and do not necessarily represent those of their affiliated organizations, or those of the publisher, the editors and the reviewers. Any product that may be evaluated in this article, or claim that may be made by its manufacturer, is not guaranteed or endorsed by the publisher.

